# Novel autosomal dominant *TNNT1* mutation causing nemaline myopathy

**DOI:** 10.1002/mgg3.325

**Published:** 2017-08-21

**Authors:** Chamindra G. Konersman, Fernande Freyermuth, Thomas L. Winder, Michael W. Lawlor, Clotilde Lagier‐Tourenne, Shailendra B. Patel

**Affiliations:** ^1^ Department of Neurosciences University of California San Diego San Diego California; ^2^ MassGeneral Institute for Neurodegenerative Disease Department of Neurology Massachusetts General Hospital Harvard Medical School Charlestown Massachusetts; ^3^ Broad Institute of Harvard University and MIT Cambridge Massachusetts; ^4^ Prevention Genetics Marshfield Wisconsin; ^5^ Division of Pediatric Pathology Department of Pathology and Laboratory Medicine and Neuroscience Research Center Medical College of Wisconsin Milwaukee Wisconsin; ^6^ Division of Endocrinology Metabolism and Clinical Nutrition Medical College of Wisconsin, and Clement J. Zablocki VAMC Milwaukee Wisconsin; ^7^ Present address: Invitae Corporation San Francisco California; ^8^ Present address: Division of Endocrinology, Diabetes and Metabolism University of Cincinnati Cincinnati Ohio

**Keywords:** Congenital myopathy, nemaline myopathy, *TNNT1*, troponin T1

## Abstract

**Background:**

Nemaline myopathy (NEM) is one of the three major forms of congenital myopathy and is characterized by diffuse muscle weakness, hypotonia, respiratory insufficiency, and the presence of nemaline rod structures on muscle biopsy. Mutations in troponin T1 (*TNNT1*) is 1 of 10 genes known to cause NEM. To date, only homozygous nonsense mutations or compound heterozygous truncating or internal deletion mutations in *TNNT1* gene have been identified in NEM. This extended family is of historical importance as some members were reported in the 1960s as initial evidence that NEM is a hereditary disorder.

**Methods:**

Proband and extended family underwent Sanger sequencing for *TNNT1*. We performed RT‐PCR and immunoblot on muscle to assess *TNNT1* RNA expression and protein levels in proband and father.

**Results:**

We report a novel heterozygous missense mutation of *TNNT1* c.311A>T (p.E104V) that segregated in an autosomal dominant fashion in a large family residing in the United States. Extensive sequencing of the other known genes for NEM failed to identify any other mutant alleles. Muscle biopsies revealed a characteristic pattern of nemaline rods and severe myofiber hypotrophy that was almost entirely restricted to the type 1 fiber population.

**Conclusion:**

This novel mutation alters a residue that is highly conserved among vertebrates. This report highlights not only a family with autosomal dominant inheritance of NEM, but that this novel mutation likely acts via a dominant negative mechanism.

## Introduction

Nemaline myopathy (NEM; MIM# 161800) is a clinically and genetically heterogeneous form of congenital myopathy characterized by muscle weakness and the presence of nemaline rods on histologic examination (Greenfield et al. 1958; Shy et al. 1963; Romero et al. 2013). NEM is one of the three major types of congenital myopathy that include nemaline myopathy, centronuclear myopathy, and core‐related myopathies. The incidence of NEM is estimated at 1:50,000 (Romero et al. 2013). The prototypic clinical features include congenital hypotonia, weakness of proximal, bulbar, facial, and neck flexor muscles that may result in death secondary to respiratory insufficiency (Romero et al. 2013). Mutations in 10 genes are currently known to cause NEM: *actin alpha 1* (*ACTA1*) (Nowak et al. 1999), *nebulin* (*NEB*) (Pelin et al. 1999), *alpha‐tropomyosin* (*TPM3*) (Laing et al. 1995a,b), *beta‐tropomyosin* (*TPM2*) (Donner et al. 2002), *troponin T1* (*TNNT1*) (Johnston et al. 2000), *cofilin‐2* (*CFL2*) (Agrawal et al. 2007), *Kelch repeat and BTB domain‐containing 13* (*KBTBD13*) (Sambuughin et al. 2010), *Kelch‐like family member 40* (*KLHL40*) (Ravenscroft et al. 2013), *Kelch‐like family member 41* (*KLHL41*) (Gupta et al. 2013), and *leiomodin‐3* (*LMOD3*) (Yuen et al. 2014). The proteins encoded by these genes are all key to the function of sarcomeric thin filaments.

*TNNT1*‐related NEM (NEM5, MIM 605355) is a recessive disorder that, until recently, was known to occur only in the Old Order Amish of Pennsylvania. In this population all patients carry a homozygous nonsense founder mutation, c.538G>T (p.E180X), resulting in a premature stop codon in exon 11 (Johnston et al. 2000). Amish nemaline myopathy presents with tremors of the jaw and lower limbs in the neonatal period that gradually subside with age, development of progressive proximal muscle contractures, diffuse atrophy, delays in gross motor development, pectus carinatum, and respiratory insufficiency by the second year of life (Johnston et al. 2000). The p.E180X mutation results in complete loss of troponin T1 in muscle (Jin et al. 2003). In 2014, van der Pol et al. described the first case of NEM5 known outside of the Old Order Amish in a Dutch family with a compound heterozygous splice site c.309+1G>A mutation and an exon 14 deletion in *TNNT1*. The c.309+1G>A mutation in splice donor site of exon 8 results in abnormal skipping of this exon and the production of a shortened mRNA transcript (van der Pol et al. 2014). Deletion of exon 8 was shown to alter the function of tropomyosin‐binding site 1 and the deletion of exon 14 is predicted to destabilize the TNNT1 protein, thereby reducing binding affinity and incorporation into the thin filament (Amarasinghe et al. 2016). The Dutch clinical phenotype was similar to Amish NEM with hypotonia, delayed gross motor milestones, progressive contractures, diffuse weakness with atrophy, absent reflexes, pectus carinatum, severe kyphosis, spinal rigidity, and absence of tremors, eventually causing respiratory insufficiency requiring home ventilation at age 2 (van der Pol et al. 2014). Marra et al. (2015) reported a second case of NEM5 in a Hispanic boy born from consanguineous parents and carrying a homozygous nonsense c.323C>G (p.S108X) mutation. This variant occurs in exon 9 and results in a truncated troponin T1 protein with 155 missing amino acids at its C‐terminus, thereby damaging tropomyosin‐binding site 1 (Amarasinghe et al. 2016). The clinical phenotype of this Hispanic male is reminiscent of the Amish NEM with hypotonia, delayed motor development without achieving crawling or independent walking and dysarthria with mild expressive language delay without cognition abnormality by 2.5 years of age (Marra et al. 2015). Other salient features include a high‐arched palate, pectus carinatum, thoracic kyphoscoliosis, hip and knee contractures, facial weakness with tented mouth, prominent head lag, prominent proximal muscle weakness, and need for noninvasive ventilation and gastrostomy tube placement by 2.5 years (Marra et al. 2015). Finally, Abdulhaq et al. described nine Palestinian patients from seven unrelated families all carrying a similar homozygous c.574_577 indel TAGTGCTGT (L203*) in *TNNT1* leading to a C‐terminal truncation of the protein which reduces affinity of troponin T1 protein to tropomyosin (Abdulhaq et al. 2015; Amarasinghe et al. 2016). The Palestinian patients' phenotype also resembled the Amish population with the exception of rigid spine with kyphosis, stiff neck, and a transient tremor (Abdulhaq et al. 2015).

All five *TNNT1* mutations described so far have involved the functionally important and evolutionarily highly conserved C‐terminal region of the troponin T1 protein (Jin et al. 1998; Johnston et al. 2000; van der Pol et al. 2014; Marra et al. 2015; Mondal and Jin 2016).

We report a novel autosomal dominantly inherited missense mutation in *TNNT1* gene (c.311A>T, p.E104V) with considerable intrafamilial clinical heterogeneity in a large family with Ashkenazi Jewish ancestry residing in the United States. This family is of historical importance since various members of the family were clinically and pathologically described by Spiro and Kennedy (1965) and Gonatas et al. (1966) as the first cases of hereditary nemaline myopathy.

## Patients and Methods

### Patient cohort

**Figure 1:**
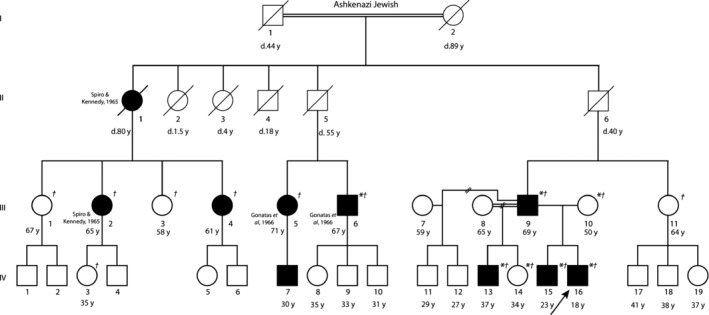
Family pedigree with current age or age at time of death. Solid black circles and squares designate affected female and male subjects, respectively, as proven by skeletal muscle biopsy or muscle weakness. Arrow indicates proband. Subject I.1 is the half‐uncle of I.2, and III.8 and III.9 are third cousins. *Examined personally by author CGK. †Genetically tested. Subjects published previously are cited.

Members of an extended family with Ashkenazi Jewish ancestry, living in distant parts of the United States, had their blood samples drawn after obtaining informed consent. Information regarding the history, clinical manifestations and rate of progression, age of onset, and genetic relationships were collected. Some of the participants were evaluated personally by one of the authors (CGK, indicated by asterisk on pedigree, Fig. 1). Clinical data were obtained either personally, by chart review, or via telephone for subjects III.1, III.2, III.3, III.4, III.5, III.6, III.9, III.10, III.11, IV.3, IV.13, IV.14, IV.15, IV.16 (Table 1). The Institutional Review Board of the Medical College of Wisconsin approved this study and all participants provided written informed consent.

**Table 1:** Clinical, genetic, pathological, and laboratory findings of affected and unaffected individuals in the extended family

Pedigree designation Age Historical publication	Mutation(s)	Pertinent clinical findings and other studies	Muscle biopsy findings
II.1 Died at age 80 Spiro and Kennedy (1965) Clinical history obtained through daughter II.2	Deceased	Inability to heel walk and waddle at age 8 Mild shoulder/pelvic girdle weakness at age 39 Intermittent use of walker and DOE at age 80 Normal DTR throughout at 39 years of age Slender build Able to ascend stairs with marked difficulty prior to death Died of a stroke and complications of heart failure at age 80	Nemaline rods present in type 1 and 2 fibers and distributed unevenly throughout sarcoplasm Qualitatively fewer rods than earlier manifesting daughter III:2
III.1 65 years	No mutation in *TNNT1* *NEB* c.8734 T>C, p.S2912P	No symptoms	
III.2 65 years Spiro and Kennedy (1965)	*TNNT1* c.311A>T, p.E104V *NEB* c.8734 T>C, p.S2912P	Lordotic posture in infancy with Gowers' sign, difficulty climbing stairs and running, waddling gait Pes cavus and inability to walk on heels at 10 years Mild DOE since teens Moderate difficulty walking prolonged distances Decreased DTR in upper extremities Mild occasional dysphagia since ˜age 50 Slender build At age 65, still ambulatory and can walk for more than 1 h per day Noticed improvement in DOE with daily sustained exercise for several hours	Nemaline rods present in type 1 and 2 fibers distributed unevenly throughout sarcoplasm
III.3 58 years	No mutation in *TNNT1* *NEB* c.8734 T>C, p.S2912P	No symptoms	
III.4 61 years	*TNNT1* c.311A>T, p.E104V *NEB* c.8734T>C, p.S2912P	Gowers' sign and difficulty running, climbing, jumping since age 5 Childhood toe walking with minor difficulty heel walking at age 54 Gradual weakness in leg but independently ambulatory at age 60 Slender build No pectus deformity High arched palate Unable to run at age 54; minor difficulty swallowing	Nemaline rods mostly present in type 1 and rarely found in type 2 fibers. Fiber type disproportion with atrophic type 1 and hypertrophic type 2 fibers.
III.5 71 years Gonatas et al. (1966)	*TNNT1* c.311A>T, p.E104V *NEB* c.8734T>C, p.S2912P	Gowers' sign in late teens Difficulty walking long distance, rising from chair, climbing stairs Slender build Pectus carinatum DTR were normal at age 18 Snoring and obstructive sleep apnea At age 67, can walk independently Unable to heel walk	Nemaline rods mostly present in type 1 and rarely found in type 2 fibers. Fiber type disproportion with atrophic type 1 and hypertrophic type 2 fibers.
III.6* 67 years Gonatas et al. (1966)	*TNNT1* c.311A>T, p.E104V *NEB* c.8734 T>C, p.S2912P	Slow runner, difficulty riding bicycle compared to peers as a teenager Mild gradual proximal muscle weakness with age High arched palate Pectus carinatum Myopathic, elongated facies DTR absent only at Achilles and brachioradialis bilaterally At age 63, no longer able to run or jump, has minor difficulty ascending stairs but can walk ˜3 miles per day No Gower's maneuver at age 63 Kyphosis; mild scoliosis since age 15 Scapular winging Slender build Able to heel walk, but mildly unsteady CK 142 (ref 39–308 IU/L)	Nemaline rods mostly present in type 1 and rarely found in type 2 fibers. Fiber type disproportion with atrophic type 1 and hypertrophic type 2 fibers. Type 1 fiber predominance.
III.9* 69 years	*TNNT1* c.311A>T, p.E104V *NEB* c.8801G>A, p.R2934H *NEB* c.8734 T>C, p.S2912P	In retrospect, noted inability to “bulk up” in his 20s (no focal muscle weakness) during weight training Very active in his youth but lean musculature With age, felt minor fatiguing in legs with prolonged exercise Elongated facies, high arched palate Normal reflexes No pectus deformities No difficulty walking on heels Minor dysphagia noted at age 65 Reported symptomatic improvement in endurance with l‐tyrosine 2 g/day for 1 year CK 176 at age 65 (ref 30–220 U/L)	Nemaline rods almost exclusively present in type 1 fibers and very rarely in type 2 fibers. Evidence of fiber type disproportion with atrophic (20–40 *μ*m) type 1 and hypertrophic (80–100 *μ*m) type 2 fibers. Type 1 fiber predominance.
III.10* 50 years	None in *TNNT1* and *NEB* genes	No symptoms or signs	
III.11 64 years	No mutation in *TNNT1* *NEB* c.8734 T>C, p.S2912P	No symptoms	
IV.3 35 years	No mutation in *TNNT1* *NEB* c.8734 T>C, p.S2912P	No symptoms	
IV.13* 37 years	*TNNT1* c.311A>T, p.E104V No mutation in *NEB*	Minor occasional tripping Minor difficulty heel walking with mild Achilles contractures bilaterally High arched palate with elongated facies No proximal muscle weakness, all DTRs present CK 57 (ref 38–174)	Biopsy slides poor quality to confirm presence of nemaline rods, but nemaline rods reported by patient.
IV.14* 34 years	No mutation in *TNNT1* *NEB* c.8801G>A, p.R2934H *NEB* c.8734 T>C, p.S2912P	No symptoms	
IV.15* 23 years	*TNNT1* c.311A>T, p.E104V No mutation in *NEB*	Mild difficulty with lower extremity weight lifting at ˜age 15 High arched palate Mild myopathic facies Reduced reflexes in upper extremities compared to lower, however absent at ankles Normal strength with ability to squat, jump, rise from kneeling position Unable to heel walk No pectus deformity No scoliosis	Nemaline rods almost exclusively in type 1 fibers and very rarely in type 2 fibers. Evidence of congenital fiber type disproportion with relatively atrophic (20–70 *μ*m) type 1 and hypertrophic (100–130 *μ*m). EM demonstrated mitochondrial inclusions but no nemaline rods (likely secondary to sampling artifact).
IV.16* Proband 18 years	*TNNT1* c.311A>T, p.E104V *NEB* c.8801G>A, p.R2934H	Fatiguing of lower extremity muscles with prolonged exercise and mild facial weakness High arched palate Elongated myopathic facies Pectus carinatum Mild scoliosis Mild waddling gait and inability to walk on heels at age 8 Slender build Mild proximal muscle weakness with poor jump and quadriceps weakness Reduced DTR in upper extremities EMG done at age 11 was myopathic Reported symptomatic improvement in endurance with l‐tyrosine 2 g/day for 3 years CK 249 (ref 30–150 IU/L)	Nemaline rods almost exclusively present in type 1 fibers. Striking fiber type disproportion with atrophic type 1 fibers and type 1 predominance

*Examined by CGK; CK, creatine kinase; N/A, not available; DOE, dyspnea on exertion; TNNT1, troponin T1 gene; NEB, nebulin gene; DTR, deep tendon reflexes; EMG, electromyography; EM, electron microscopy.

### Mutation screening

Genomic DNA was extracted as per standard protocols from blood in all 15 individuals who agreed to participate. The proband underwent Sanger sequencing of full coding regions and ~50 bases of flanking noncoding sequences of nine of the NEM‐related genes (*ACTA1* [NM_001100.3], *CFL2* [NM_021914.7], *KLHL41* [NM_006063.2], *KBTBD13* [NM_001101362.2], *KLHL40* [NM_152393.3], *NEB* [NM_001271208.1], *TNNT1* [NM_003283.4], *TPM2* [NM_003289.3], *TPM3* [NM_152263.3]). *LMOD3* was not evaluated as it was identified as a cause of NEM after this genetic analysis had been completed (Yuen et al. 2014). The proband also underwent analysis of *NEB* exon 55 deletion using appropriate primers that flank or lie within the deleted exon (Anderson et al. 2004). To determine which variants found in the proband segregated with disease, the remaining 14 subjects were subsequently analyzed by PCR amplification and sequencing for *TNNT1* exon 9 and *NEB* exon 62. Primer sequences used are available on request.

### Muscle pathology

Vastus lateralis open muscle biopsies of three affected family members (Fig. 1; subjects IV.16 [proband], IV.15, III.9) were analyzed at time of presentation or if genetic analysis indicated presence of the suspected segregating mutation. One specimen was frozen in isopentane precooled by liquid nitrogen and a small piece of muscle was fixed in 4% glutaraldehyde. Standard techniques were applied for hematoxylin and eosin (H&E) and enzyme histochemical staining. Biopsies from subjects III.9 and IV.15 underwent further structural analysis via electron microscopy using standard techniques at the Medical College of Wisconsin (MCW) Electron Microscopy Core Facility.

### RNA isolation and expression analysis via reverse transcription and PCR

Total RNA was isolated from human muscle tissue using Trizol reagent (Thermo Fisher Scientific, Carlsbad, CA, USA). Genomic DNA was removed from RNA by incubation with TURBO DNA‐*free* kit (Thermo Fischer Scientific, Vilnius, Lithuania) at 37°C for 30 min. Using SuperScript III Reverse Transcriptase (Thermo Fisher Scientific, Carlsbad, CA, USA), 400 ng of total RNA was reverse transcribed to generate cDNA. PCR was performed with Taq polymerase, one denaturation step at 98°C for 5 min, 28 cycles of amplification at 98°C for 15 sec, 67°C for 30 sec, 72°C for 1 min, and a final step at 72°C for 5 min using the specific human primers for *TNNT1* exon 5 (forward: GGCTCAGCCTCAAGATTCAC, reverse: TCCAGCAGGTCTTTCTCCAT), for *TNNT1* exon 9 (forward: TGGAGCTGCAGACACTCATC; reverse: TTACCACGCTTCTGTTCTGC), and for *TNNT1* exon 12 (forward: GCAGAACAGAAGCGTGGTAA, reverse: GCCATCAGGTCGAACTTCTC). The PCR products were analyzed by electrophoresis with 10% polyacrylamide native gel, stained with SYBR gold nucleic acid gel stain (Thermo Fisher Scientific, Eugene, OR, USA, 1:10,000) and visualized with gel imaging system (Bio‐Rad, Hercules, CA, USA). The imaging quantification of each PCR product was performed using ImageJ quantification.

### Western blotting of TNNT1 protein

Total protein extracts isolated by Trizol reagent (Thermo Fisher) from biopsied vastus lateralis muscle from subjects III.9 (biopsied at age 65), IV.16 (biopsied at age 12), an age‐matched 12‐year‐old healthy male control, and a 61‐year‐old healthy female control were obtained. Muscle biopsies from subjects III.9 and IV.16 were obtained under an IRB‐approved protocol through the Congenital Muscle Disease Tissue Repository (http://www.mcw.edu/Congenital-Muscle-Disease-CMD-Tissue-Repository.htm). Control biopsies were obtained from Dr. Denise Malicki and Dr. Karra Jones under an IRB‐approved research protocol at UCSD. The protein samples were mixed with 5X Laemmli sample buffer and denatured at 95°C for 5 min. The muscle protein extracts were resolved on 14% Laemmli gel with an acrylamide to *bis*‐acrylamide ratio 180:1 and transferred onto PVDF membrane. After blocking with 5% nonfat dry milk (NFM) in Tris‐buffered saline (TBS, 150 mmol/L NaCl, 50 mmol/L Tris‐HCl, pH 7.5), the membrane was either incubated with mouse monoclonal antibody anti‐slow troponin T (clone CT3, Santa Cruz, sc‐20025, 1:500) or with rabbit monoclonal anti‐GAPDH (clone 14C10, Cell Signaling, 2118, 1:2000). Then, the membrane was washed three times and incubated with anti‐mouse or anti‐rabbit peroxidase antibody (GE Healthcare Life Sciences, 1:10,000) for 1 h in TBS‐5% NFM, followed by revelation using ECL chemiluminescence system (Thermo Fisher Scientific, Rockford, IL, USA).

## Results

### Clinical features

The proband, an 18‐year‐old Ashkenazi Jewish male (Fig. 1, Table 1, IV.16) born to nonconsanguineous parents, presented at 8 years of age with mild facial and palatal weakness and minor fatigue of his lower extremity muscles when swimming. He exhibited very mild progression of proximal weakness with age, manifesting as fatigue in the legs during prolonged exercise. Examination showed a slender build, high‐arched palate, elongated facies, pectus carinatum, mild scoliosis, mild bilateral isolated Achilles contractures, myopathic facies, mild quadriceps and ankle dorsiflexion weakness, poor jump, and reduced reflexes only in the upper extremities. Of note, his intellect, extraocular muscle function, cardiac function, and breathing and swallowing functions were normal. EMG performed at age 11 years demonstrated normal nerve conductions with evidence of a nonirritable myopathy in proximal and distal lower extremity muscles (data not shown). Creatine kinase (CK) was mildly elevated at 249 (reference 30–150 IU/L).

The family history of the proband revealed multiple cases of myopathy in three different generations and a history of consanguinity with individual I.1 being the half‐uncle of I.2 and individuals III.8 and III.9 being third cousins (Fig. 1). Table 1 summarizes the age of onset, presenting symptoms, associated clinical features, and relevant studies of studied members of this large family.

Symptoms in the proband's brother (IV.15) and father (III.9) started at 15 and 20 years of age, respectively, both of whom represented milder phenotypes than the proband with self‐reported complaints of poorer endurance in the legs during weight training compared to their peers (Fig. 1 and Table 1). Both exhibited no focal weakness and were fairly athletic, although mild myopathic facies with high‐arched palates were noted. The proband's brother could not heel walk and had reduced reflexes in the upper extremities. The proband's father could heel walk and had intact reflexes, but developed intermittent mild dysphagia at 65 years of age with normal levels of creatine kinase (CK) at 176 IU/L (reference 30–220 IU/L).

A broad spectrum of clinical heterogeneity was observed in this family. The earliest onset of weakness was in childhood ranging from ages 5 to 10 years for subjects IV.16 (proband; onset at age 8 years), II.1 (onset at age 8 years), III.2 (onset at age 10 years), III.4 (onset at age 5 years) ranging from a Gower's maneuver for subject III.4 to mild waddling gait and inability to walk on heels for subject III.2 (Table 1). The oldest affected member of the family, subject II.2 died at age 80 years from presumably unrelated stroke and complications of heart failure, despite having onset of muscle weakness at 8 years of age (Table 1). The overall pattern of weakness was proximal, manifesting as a Trendelenburg gait, Gower's maneuver, or difficulty in performing repeated exercises that require proximal muscle strength. However, isolated ankle dorsiflexion weakness was a common early distal manifestation. Several subjects exhibited pectus carinatum (III.5, III.6, IV.16; Table 1). Mild scoliosis or kyphosis was seen in IV.16 and III.6 with onset in the teens (Table 1). High‐arched palate and/or myopathic elongated facies was seen in subjects IV.16, III.4, III.6, III.9, IV.13, and IV.15 (Table 1). The weakness progressed slowly over time and the most severely affected members were still independently ambulatory in their 50–60s (III.2, II.4, III.5) and subject II.1 was ambulatory over short distances with a walker until her time of death at age 80 years (Table 1). Subjects IV.16 and III.9 reported subjective symptomatic improvement in exercise endurance after taking l‐tyrosine 2 g/day for at least 1 year (Table 1).

Subject IV.7 was unavailable for clinical or molecular testing, however, his mother reported weakness (Table 1). Subject II.5 died at the age of 55 years of encephalitis and II.6 died at the age of 40 years from glomerulonephritis prior to developing any significant symptoms (Table 1).

### Muscle pathology

**Figure 2:**
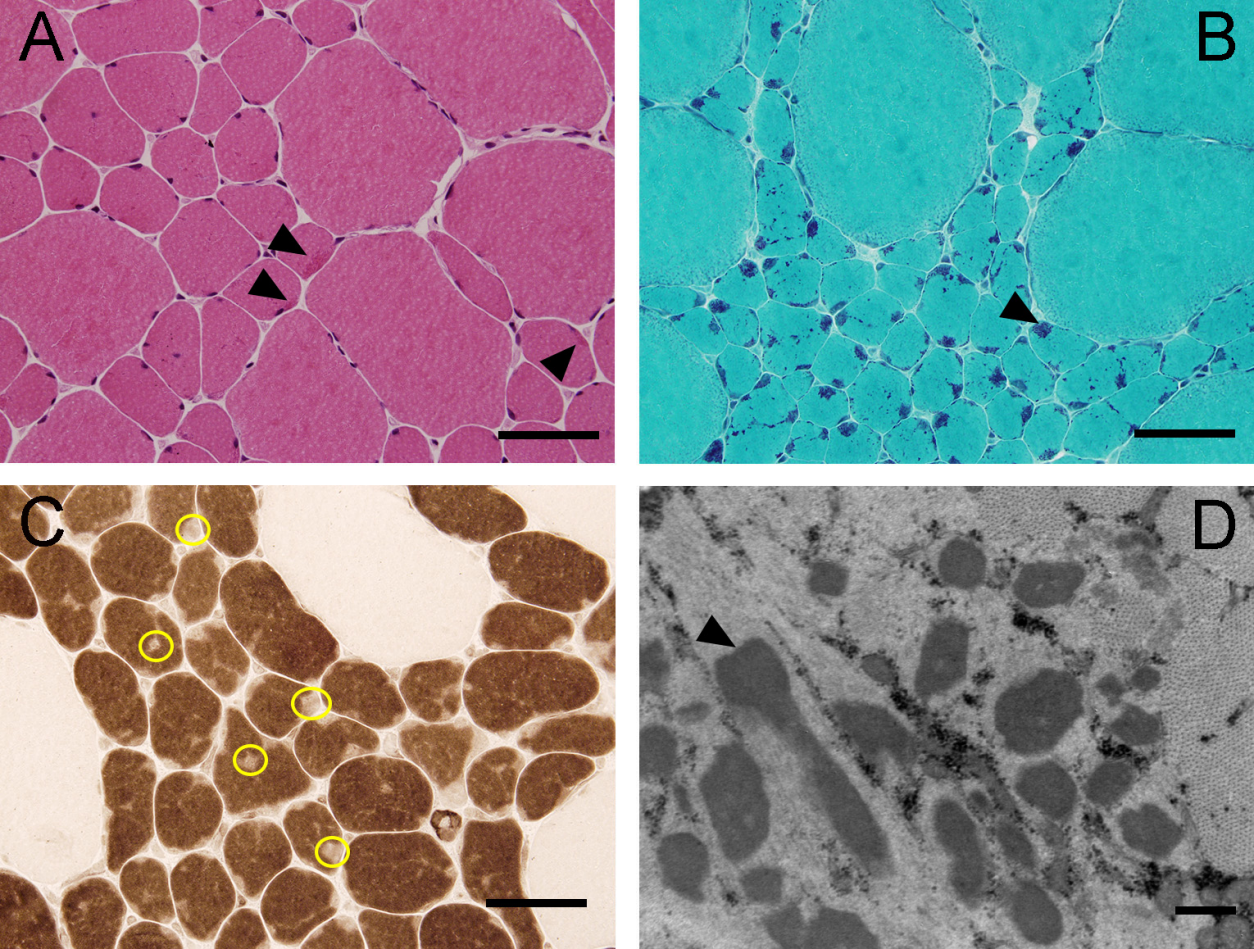
Pathological analysis of quadriceps muscle (A–D) reveals considerable fiber size disproportion and presence of nemaline rods. H&E (A) demonstrates clusters of atrophic type 1 fibers and hypertrophic type 2 fibers with nemaline rods as dark red inclusions (arrowheads). These same inclusions are better visualized on Gomori trichrome (B) as purple/blue inclusions (arrowhead) almost exclusively localized to the atrophic type 1 fibers. The ATPase stain (C) at pH 4.3 demonstrates absence of staining in the location of rods (yellow circles) in the hypotrophic type 1 fibers (dark staining). Electron microscopy (D) demonstrates the electron dense nemaline rods (arrowhead). Scale bar = 50 *μ*m for panels A–C and 500 nm for panel D.

Skeletal muscle biopsies of the vastus lateralis in subjects III.9, IV.15, and IV.16 identified severe type 1 fiber hypotrophy (diameter 10–70 microns), with numerous red/purple‐staining rod‐like structures on Gomori trichrome exclusively in type 1 fibers (Fig. 2A–C, muscle histopathology). These inclusions were distributed unevenly throughout the sarcoplasm. Type 2 fibers in the specimens were hypertrophic (diameter 90–130 microns), and there was type 1 fibers predominance (ranging from 60% to 90% type 1 fibers) across all three biopsies. Mild increased internal nucleation was present. There were no cores, inflammation, or increased endomysial connective tissue. Electron microscopy for III.9 and IV.15 confirmed the presence of electron dense bodies consistent with nemaline rods in the former, however, not in the latter (likely due to sampling; Fig. 2D). Unlike the patients studied here (III.9, IV.15, and IV.16), histopathology of subjects II.1 and III.2 published in 1965 demonstrated presence of nemaline rods in both type 1 and 2 fibers (Fig. 1, Table 1) (Spiro and Kennedy 1965). In contrast, muscle pathology for subjects III.5 and III.6 revealed nemaline rods mostly in type 1 and rarely in type 2 fibers with type 2 fiber hypertrophy, recapitulating our findings (Fig. 1, Table 1) (Spiro and Kennedy 1965; Gonatas et al. 1966).

### Mutation detection

**Figure 3:**
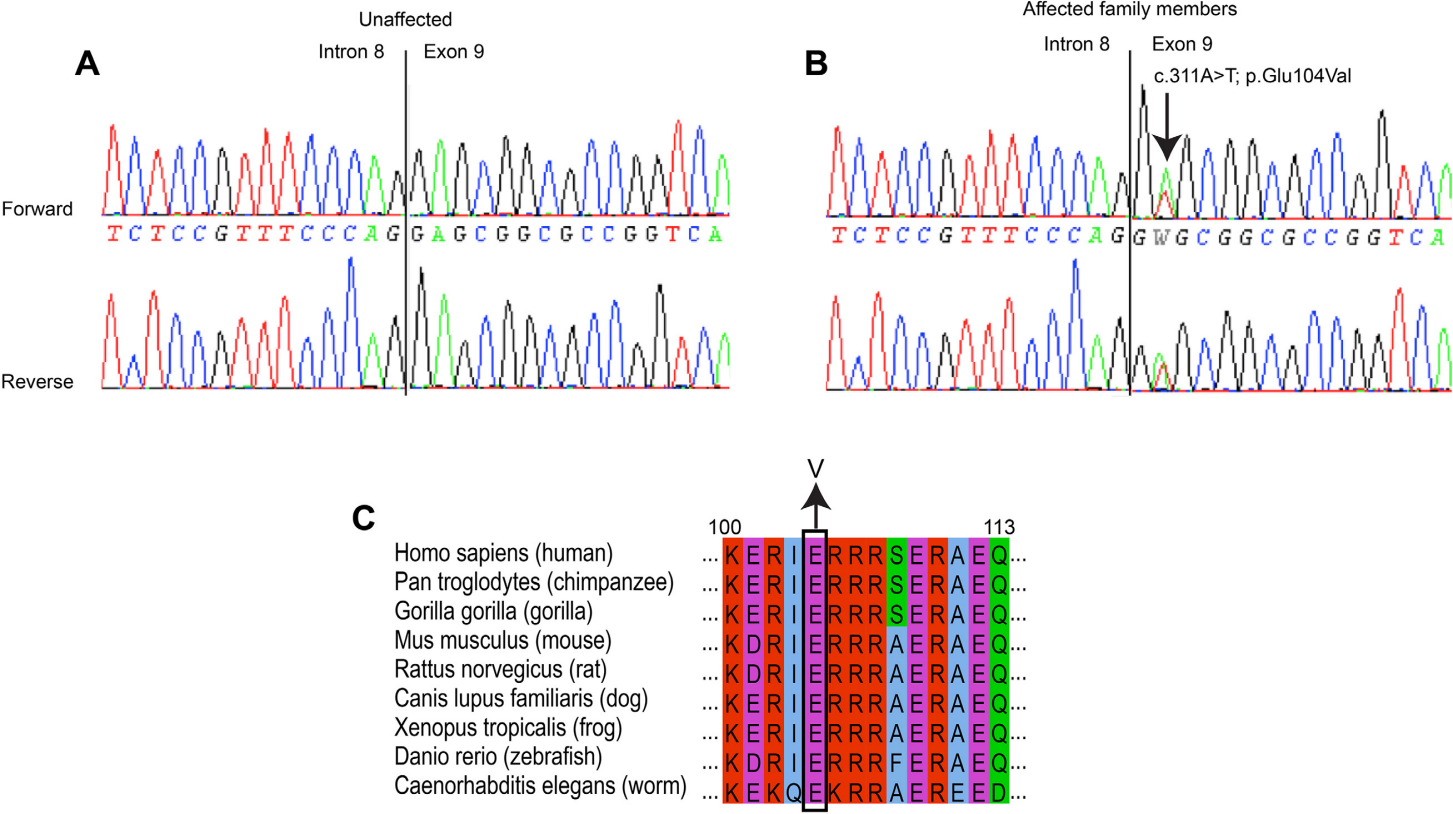
Electropherogram of *TNNT1* mutation in unaffected (A) and affected (B) family member showing a c.311A>T (p.E104V) in the latter. (C) Alignment of troponin T1 protein showing complete conservation of residue 104 across species.

Genetic testing in the proband demonstrated the presence of a novel heterozygous missense variants of unknown clinical significance in exon 9 of the gene *TNNT1* c.311A>T (p.E104V) (NM_003283.4) (Fig. 3A and B, electropherogram). The *TNNT1* c.311A>T change is located at the 3′ splicing site of the evolutionarily conserved exon 9 among vertebrates (Jin et al. 1998). This variant was absent from the NHLBI EVS and the 1000 Genomes database (Genomes Project C, Abecasis, et al., 2012) and the Exome Aggregation Consortium (ExAC, Cambridge, MA; URL: http://exac.broadinstitute.org). Furthermore, molecular analysis in the proband revealed no significant sequence variants or unknown variants in *ACTA1*,* NEB*,* CFL2*,* TPM2*,* TPM3*,* KBTD13*,* KLHL40*, and *KLHL41* genes. PCR detection using primers that flank the documented deletion of *NEB* exon 55, an Ashkenazi Jewish founder mutation, was also negative (Anderson et al. 2004). Subjects III.2, III.4, III.5, III.6, and III.9, all of whom have known nemaline rods on pathology, also have the *TNNT1* c.311A>T (p.E104V) change (Table 1). Subject II.2 was deceased at the time of this study, thus mutation analysis was not possible. None of the six unaffected family members tested had the *TNNT1* c.311A>T mutation. Two variants already reported in individuals not affected with congenital myopathy, *NEB* c.8734 T>C (p.S2912P) and c.8801G>A (p.R2934H), were found in several individuals of the family but did not segregated with disease. Therefore, only the *TNNT1* mutation segregated with clinical and pathological phenotype.

The glutamic acid residue at position 104 is highly conserved in human slow skeletal muscle troponin T1, cardiac troponin T2, and fast skeletal muscle troponin T3 (Jin and Chong 2010; Wei and Jin 2016). The E104 is located in the highly conserved tropomyosin‐binding site 1 region among vertebrates of the troponin T1 protein, suggesting its importance in the structure and/or the function of troponin T1 (Amarasinghe et al. 2016; Mondal and Jin 2016; Wei and Jin 2016).

### Splicing and protein level analysis

**Figure 4:**
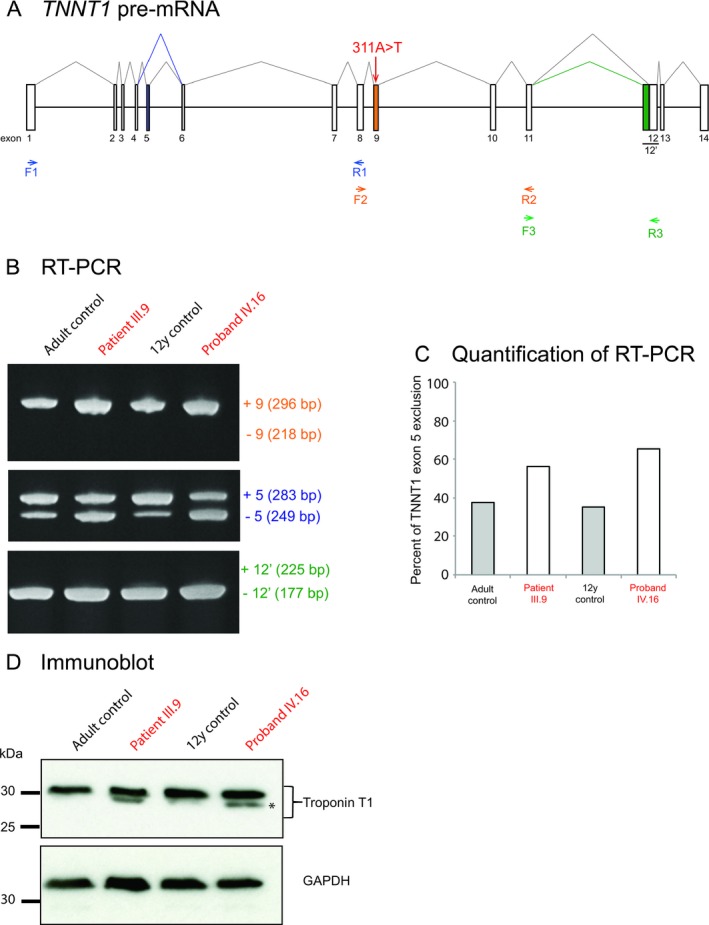
Analysis of *TNNT1* mRNA splicing and troponin T1 protein expression in nemaline myopathy patients harboring 311A>T mutation. (A) Schematic representation of the *TNNT1* pre‐mRNA. The 311A>T mutation is located in the 3′ splicing site of the constitutive exon 9 of *TNNT1* gene, with the boxes representing the exons and the black lines representing the introns. The alternative exon 5, the constitutive exon 9, and the alternative exon 12′ (partial retention of intron 11) are shown in blue, orange, and green boxes, respectively. Forward (F1, F2, F3) and reverse primers (R1, R2, R3) used for the detection of the *TNNT1* splicing isoforms by reverse transcriptase RT‐PCR are represented with arrows. Primers F1 and R1, F2 and R2, F3 and R3 were used to analyze the splicing of the exons 5, 9, and 12′, respectively. (B) RT‐PCR analysis of the splicing of *TNNT1* exon 9 (upper panel), exon 5 (middle panel), and exon 12′ (lower panel) in vastus lateralis muscle from control (black) and affected subjects IV.16 and III.9 (red) carrying the 311A>T mutation. Exons 5, 9, and 12′ inclusions (+5, +9, +12′), exclusions (−5, −9, −12′), and expected sizes of the PCR products are indicated on the right of each panel. (C) Quantification of *TNNT1* transcripts with exclusion of exon 5 in vastus lateralis muscle from control and affected subjects IV.16 and III.9 carrying the 311A>T mutation. The percentage of the exon exclusion was calculated by the ratio of the intensity of the upper PCR product relative to the sum of two products on acrylamide gel. (D) Total protein extracted from vastus lateralis muscle biopsies was analyzed by immunoblotting for troponin T1 protein with the monoclonal CT3 antibody. Full‐length protein is observed at the expected molecular weight. A low‐molecular‐weight troponin T1 isoform reported to result from mRNA with exon 5 skipping (Larsson et al. 2008) is detected (asterisk). GAPDH (glyceraldehyde‐3‐phosphate dehydrogenase) was used as a loading control.

*TNNT1* consists of 14 exons and encodes for a 30‐ to 35‐kDa protein with variable N‐terminal regions and conserved middle and C‐terminal regions (Jin et al. 2003). The mutation c.311A>T is located in exon 9, two nucleotides from the intron 8–exon 9 junction at the splice acceptor site (Figs. 3B, 4A). We first determined whether the mutation was associated with abnormal splicing of exon 9 in RNA extracted from vastus lateralis muscle biopsies of the proband (IV.16), affected father (III.9), and two age‐matched controls (Fig. 4B). By reverse transcriptase PCR using primers in exons 8 and 11, we found that *TNNT1* exon 9 was normally included in *TNNT1* transcripts from both patients (Fig. 4B; upper panel). We then examined the inclusion of exon 5 and exon 12′ (a longer version of exon 12) which were reported to be alternatively spliced exons (Gahlmann et al. 1987; Samson et al. 1994; Jin et al. 1998; Zhang et al. 2014). Indeed, previous work reported that resistance training increased expression of *TNNT1* mRNA isoforms without exons 5 and 12′ in vastus lateralis, whereas a sedentary lifestyle increased the inclusion of these exons (Zhang et al. 2014). In addition, Larsson et al. determined that expression of the *TNNT1* isoform missing exon 5 was increased in the demyelinating forms of Charcot–Marie–Tooth (CMT type 1) but not in axonal form of the disease (CMT type 2) (Larsson et al. 2008). We did not observe any inclusion of exon 12′ in vastus lateralis muscle biopsies of the proband (IV.16) and his affected father III.9 (Fig. 4B; lower panel). In contrast, transcripts without exon 5 were increased in both patients compared to age‐matched controls (Fig. 4B, middle panel and Fig. 4C). Consistent with this result, immunoblot of proteins extracted from vastus lateralis muscle biopsies identified increased levels of a low‐molecular‐weight band in both patients (Fig. 4D). This short isoform of troponin T1 protein was previously shown to result from skipping of exon 5 (Larsson et al. 2008). Notably, the c.311A>T (p.E104V) was not associated with reduced level of the protein in muscles from patients compared to controls (Fig. 4D).

## Discussion

This study describes the first *TNNT1* mutation that transmits in an autosomal dominant fashion to cause nemaline myopathy. *TNNT1* encodes troponin T type 1, which is exclusively found in slow skeletal muscle (type 1 fibers) and serves to anchor the troponin complex (along with troponin C and I) onto the tropomyosin–actin thin filaments (Nadal‐Ginard and Mahdavi 1989; Jin et al. 2003; Wei et al. 2014). Three homologous genes encode different isoforms of troponin T in specific tissues: slow skeletal muscle troponin T (*TNNT1*), fast skeletal muscle troponin T (*TNNT3*), and cardiac troponin T (*TNNT2*) with alternative splicing of each gene conferring protein variants with slightly different functional abilities (Samson et al. 1994; Jin et al. 2003; Wei et al. 2014). Troponin T1 regulates the conformational changes in the thin filaments during excitation–contraction–coupling of slow skeletal muscle (Wei et al. 2014; Wei and Jin 2016).

All of the previously described mutations were autosomal recessive cases of *TNNT1*‐related NEM (NEM5) (Johnston et al. 2000; van der Pol et al. 2014; Abdulhaq et al. 2015; Marra et al. 2015). The *TNNT1* c.311A>T nucleotide substitution predicts a p.E104V missense mutation which alters the nature of the amino acid from a polar to a nonpolar residue and is predicted to be pathogenic by polyphen (Adzhubei et al. 2010). The amino terminal segment undergoes alternative splicing, with studies indicating that although this region does not bind any other thin filament protein, it plays a regulatory role in the conformational changes in troponin T1, thus modulating muscle contraction and interactions with the myofilament (Jin et al. 1998; Amarasinghe et al. 2016). In contrast to the previously described recessive *TNNT1* mutations resulting in truncation or reduction of troponin T1 protein levels, we describe the first heterozygous missense mutation with evidence of intact (albeit apparently functionally altered) TNNT1 protein. Levels of troponin T1 were not affected by the mutation, and the *TNNT1* c.311A>T mutation located at the second nucleotide position of exon 9 did not alter the constitutive splicing of *TNNT1* exon 9 containing the mutation. Troponin T1 binds tropomyosin to link the troponin complex to actin via two tropomyosin binding sites present in the highly conserved regions corresponding to residues 64 to 108 and residues 180 to 204, respectively (Jin and Chong 2010; Amarasinghe et al. 2016; Mondal and Jin 2016). The p.E104 residue is located within tropomyosin‐binding site 1 (Jin and Chong 2010; Mondal and Jin 2016). We hypothesize that the mutation of a glutamic acid residue into valine at this particular position can affect the affinity of troponin T1 for tropomyosin.

A low‐molecular‐weight (LMW) troponin T1 protein isoform that results from alternative splicing of exon 5 of *TNNT1* (Jin et al. 1998; Zhang et al. 2014) was observed in both the affected proband and his father. The shorter troponin T1 is developmentally regulated: troponin T1 LMW is absent in the fetus, appears in term the newborn, and decreases with age in human quadriceps muscle (Jin et al. 1998; Zhang et al. 2014). Larsson et al. (2008) determined that expression of the LMW troponin T1 was increased in the demyelinating forms of Charcot–Marie–Tooth (CMT) type 1, presumably due to compensatory overuse of the muscle, in the setting of a reinnervating and intact axon, in comparison to CMT type 2, which has axonal destruction. We hypothesize that the shorter band identified on the immunoblot from the affected individuals could represent an upregulation of LMW TNNT1 as a physiological adaptation in patients carrying the *TNNT1* c.311A>T mutation. If this alternative splicing is a physiological response to nemaline myopathy in patients with the c.311A>T mutation, further investigation is necessary to determine whether this mechanism extends to other *TNNT1* mutations or other NEM‐related gene mutations.

Clinically, the *TNNT1* c.311A>T heterozygous missense autosomal dominant mutation results in a mild phenotype with considerable clinical heterogeneity, whereas the recessive truncating and internal deletion mutations with loss of function are associated with severe, early‐onset phenotypes (Mondal and Jin 2016). Variable expression is a common feature of autosomal dominant diseases (Strachan and Read 2011). In our family, severity ranged from mild difficulty with weight‐training exercises to a Gower's maneuver in early childhood (age 8). While the severe recessive mutations resulted in respiratory insufficiency and ventilatory assistance by 2 years of age, none of the members of this family have required ventilatory assistance even into their late 60s. Pectus carinatum, a common finding in individuals with the autosomal recessive form of the disease, is found in several members of this family and seem to correlate with increased severity. Although nonspecific, blunted upper extremity reflexes, high‐arched palate, ankle dorsiflexion weakness resulting in poor heel walk, and mild myopathic facies were the most common clinical hallmarks of our affected subjects. The impact on life span appears fairly negligible in this family, since multiple affected family members are independently ambulatory into in their 60s and subject II.1 survived to 80 years of age despite having mild symptoms since childhood. Although contractures were mild and limited to the Achilles tendon in this cohort, they were progressive and present proximally in the recessive forms of the disease. Tremors were characteristically absent in our cohort.

The fiber type disproportion found on most of the muscle biopsies suggests that the abnormality more profoundly impacts type 1 fibers. This is not surprising, as troponin T1 localizes to the type 1 muscle fibers and Wei et al. (2014) demonstrated that *TNNT1* deficiency results in approximately 40% reduction in cross‐sectional area of type 1 fibers. While the previous reports in the Amish and Dutch NEM5 families recapitulate the fiber type disproportion with type 1 hypotrophy, it is unclear whether the nemaline rods are primarily restricted to type 1 fibers in all individuals with TNNT1 mutations (Johnston et al. 2000; van der Pol et al. 2014). Marra et al. described the presence of nemaline rods in roughly 80% of type 1 and 2 fibers in their case study (Marra et al. 2015). The same pathologic finding of fiber type disproportion with atrophic type 1 fibers has been described in patients with mutations in TPM3, another protein known to cause congenital myopathy and primarily expressed in slow skeletal muscle (Laing et al. 1995; Lawlor et al. 2010; Ottenheijm et al. 2011; Marttila et al. 2014). This report illustrates that the newly identified *TNNT1* c.311A>T mutation can produce NEM with hypotrophy and nemaline rods restricted to the type 1 fiber population, and such cases should prompt genetic screening for both the *TNNT1* and *TPM3* genes in patients. The type 2 fibers in the three cases reported here display marked hypertrophy, which we presume is a compensatory adaptation to hypotrophic type 1 fibers. This compensatory hypertrophy may mitigate symptomatic weakness and suggest a role for exercise or hypertrophic agents in the management of these patients.

Three members of the family (III.9, IV.15, IV.16) reported subjective improvement in endurance after taking l‐tyrosine 2–3 g/day for several months. Ryan et al. reported improved sialorrhea and muscle strength in infants with *ACTA1* nemaline myopathy, presumably due to increased peripheral catecholamine synthesis improving sympathetically mediated salivary gland function (Ryan et al. 2008). Of note, other sympathetic systemic features, such as tachycardia and hypertension, did not occur in the Australian cohort (Ryan et al. 2008) or in our patients.

*TNNT1* appears to be emerging as a causative gene for both recessive and dominant nemaline myopathy outside the Old Order Amish population. A pattern of myofiber hypotrophy and nemaline rods restricted to type 1 fibers was specifically seen in muscle biopsies of these *TNNT1* missense mutant patients. In addition to classic infantile NEM, we propose that *TNNT1* should be considered in mild cases with nemaline rods and an autosomal dominant pattern of inheritance.

## Conflict of Interest

Dr. Lawlor is a member of advisory boards for Audentes Therapeutics and receives research support from Audentes Therapeutics, Solid GT, and Demeter Therapeutics. Drs. Lawlor and Konersman were consultants for Sarepta Therapeutics at the time that this work was performed. The remaining authors declare no conflicts of interest.
